# *Mycobacterium bovis* BCG as immunostimulating agent prevents the severe form of chronic experimental Chagas disease

**DOI:** 10.3389/fimmu.2024.1380049

**Published:** 2024-03-21

**Authors:** Minerva Arce-Fonseca, Dulce Mata-Espinosa, Alberto Aranda-Fraustro, José Luis Rosales-Encina, Mario Alberto Flores-Valdez, Olivia Rodríguez-Morales

**Affiliations:** ^1^ Laboratory of Molecular Immunology and Proteomics, Department of Molecular Biology, Instituto Nacional de Cardiología Ignacio Chávez, Mexico City, Mexico; ^2^ Laboratory of Experimental Pathology, Instituto Nacional de Ciencias Médicas y Nutrición Salvador Zubirán, Mexico City, Mexico; ^3^ Department of Pathology, Instituto Nacional de Cardiología Ignacio Chávez, Mexico City, Mexico; ^4^ Laboratory of Molecular Biology, Department of Infectomics and Molecular Pathogenesis, Centro de Investigación y de Estudios Avanzados del Instituto Politécnico Nacional, Mexico City, Mexico; ^5^ Biotecnología Médica y Farmacéutica, Centro de Investigación y Asistencia en Tecnología y Diseño del Estado de Jalisco, A. C., Guadalajara, Mexico

**Keywords:** Chagas disease, *Trypanosoma cruzi*, BCG, vaccination, trained/nonspecific immunity, immunomodulator

## Abstract

**Introduction:**

There is currently no vaccine against Chagas disease (ChD), and the medications available confer multiple side effects. *Mycobacterium bovis* Bacillus Calmette–Guérin (BCG) produces balanced Th1, Th2, and Th17 modulatory immune responses and has improved efficacy in controlling chronic infections through nonspecific immunity. We aimed to improve the response to infection by inducing a stronger immune response and greater protection against the parasite by trained immunity.

**Methods:**

BALB/c mice were immunized with BCG subcutaneously, and 60 days later, they were infected with *Trypanosoma cruzi* intraperitoneally. An evaluation of the progression of the disease from the acute to the chronic stage, analyzing various aspects such as parasitemia, survival, clinical status, and humoral and cellular immune response, as well as the appearance of visceral megas and the histopathological description of target organs, was performed.

**Results:**

Vaccination reduced parasitemia by 70%, and 100% survival was achieved in the acute stage; although the presentation of clinical signs was reduced, there was no increase in the antibody titer or in the differential production of the isotypes.

**Conclusion:**

Serum cytokine production indicated a proinflammatory response in infected animals, while in those who received BCG, the response was balanced by inducing Th1/Th2-type cytokines, with a better prognosis of the disease in the chronic stage.

## 1 Introduction

Chagas disease (ChD), also known as American trypanosomiasis, is caused by *Trypanosoma cruzi*, a hemoflagellate parasite that is transmitted through various species of blood-sucking insects, mainly in endemic areas. This parasitosis is a multisystem disorder that can affect the cardiovascular, digestive, and central nervous systems (1).

Approximately 70 million people are at risk of contracting ChD worldwide; it is estimated that six to seven million people are infected and 30,000 new cases are registered annually in the American continent (2). Formerly, ChD was restricted to areas of poverty and marginalization since dwellings in precarious conditions made of adobe, palm, wood, or sheet metal along with the presence of domestic and farm animals constitute a good niche for the presence of triatomine insects that act as its vectors (3). However, recent internal migration from rural to urban areas, congenital transmission, and blood donation have led to the spread of the disease to previously unaffected regions worldwide (4). Mexico ranks as the third country with the highest prevalence of ChD in America, after Brazil and Argentina, and after Bolivia as the country with the highest number of cases (5).

The clinical manifestations of ChD can range from very mild and even asymptomatic infection (70% of cases) to irreversible and very serious disease (30% of cases) (6). Only two drugs are available—nifurtimox and benznidazole—for the treatment of this disease, which are effective only in the acute phase or in the early stage of asymptomatic chronic phase. This has led researchers around the world to focus on controlling transmission and searching for more efficient and less toxic pharmacological or alternative treatments as well as developing prophylactic and therapeutic vaccines. However, to date, no candidate has been advanced to a clinical phase trial (7).

Bacillus Calmette–Guérin (BCG) consists of several attenuated strains of *Mycobacterium bovis* used worldwide as a neonatal vaccine against severe forms of childhood tuberculosis (TB) (8). It has been shown that, despite its genomic similarities to *Mycobacterium tuberculosis*, its side effects are minimal, and the risk of developing BCGosis is very low. BCG has been found to provide nonspecific protection against a number of infectious and non-infectious diseases (9). Further to this, BCG doses are relatively easy and inexpensive to produce. They are relatively heat stable and act as a self-adjuvant to induce innate and adaptive immune responses with protective or beneficial effects in different pathologies such as tuberculosis, leprosy, leishmaniasis, malaria, and some types of cancer (10–13). BCG has also been used as a multivalent vaccine vehicle for other human pathogens (14–17).

Regarding ChD, it has been reported that BCG vaccination induced resistance to the challenge of infection with *T. cruzi* in a murine model and inhibited the parasite’s reproduction rate in the BCG-activated macrophages (18) or that it caused a partial but significant parasitemia reduction in mice challenged with blood trypomastigotes of *T. cruzi* (19); however, the controversial results found by other authors describe that BCG immunization did not induce resistance to infection in mice even though cultured macrophages did (20). It has no effect on parasitemia and longevity of mice inoculated with *T. cruzi* (21). A significant decrease in parasitemia could not be induced in mice that were BCG-immunized and *T. cruzi*-infected (22); therefore, we contend that the effect of BCG on *T. cruzi* infections merits further investigation, as in fact it has been shown that the BCG vaccine promotes a better clinical and immunological profile of chronic chagasic cardiomyopathy associated with less cardiac involvement (23).

On the other hand, works that address the issue of trained immunity have also been reported (23–27), which consists of the ability of innate immune cells, once exposed to a first pathogenic agent (or vaccine), to undergo a process of metabolic and epigenetic reprogramming that prepares them to respond better to a second, unrelated/nonspecific infection.

Considering the well-known effects of BCG on the immune response, vaccination with this strain may be able to promote a better course of ChD. For this reason and the previous reports suggesting a potential benefit of BCG on ChD, interest arose in exploring the cardioprotective effect of *Mycobacterium bovis* BCG as an immunomodulator against *T. cruzi* in experimentally infected mice throughout the entire evolution of the disease (acute and chronic stages). Therefore, the aim of this study was to explore whether nonspecific/trained immunity induces a response against acute and chronic *T. cruzi* infection.

## 2 Materials and methods

### 2.1 Experimental animals

A total of 40 6- to 8-week-old BALB/c female mice from the Laboratory Animal Production and Experimentation Unit of the Center for Research and Advanced Studies of the National Polytechnic Institute (UPEAL-CINVESTAV, Mexico City, Mexico) were used. The animals were divided into four groups with five mice each (**Table 1**): HEALTHY—unvaccinated and uninfected mice, *Tc*—*T. cruzi*-infected mice, BCG-i—mice immunized with *Mycobacterium bovis* BCG Pasteur strain and uninfected, and BCG/*Tc*—BCG-immunized and *T. cruzi*-infected mice. The experiments were carried out in duplicate to confirm the reproducibility of our findings. All mice were housed with 12/12-h light/dark cycles at 24°C–26°C temperature conditions, and with food and water *ad libitum*. All procedures were carried out in accordance with the guidelines of the Norma Oficial Mexicana NOM-062-ZOO-1999 Technical Specifications for the Production, Care, and Use of Laboratory Animals.

**Table 1:** Description of the experimental groups.

Group (*n* = 5)	BCG strain for immunization (2 × 10^4^ CFU/mouse)	Infection (225 blood trypomastigotes/mouse)^a^
HEALTHY	None	No
*Tc*	None	Yes
BCG-i	*M. bovis* BCG Pasteur strain ATCC 35734	No
BCG/*Tc*	*M. bovis* BCG Pasteur strain ATCC 35734	Yes

a Ninoa *T. cruzi* strain (MHOM/MX/1994/Ninoa).

### 2.2 BCG culture

Vaccine doses were obtained under appropriate management conditions for *Mycobacterium bovis* BCG Pasteur ATCC 35734 strain (referred to as BCG for short) as described previously. Briefly, BCG was cultured in Middlebrook 7H9 broth supplemented with 0.5% glycerol, 0.05% Tyloxapol, 10% OADC enrichment medium (containing per liter: 0.85 g sodium chloride; serum albumin fraction V, 5 g; dextrose 2 g; catalase 0.002 g; oleic acid, 0.05 g), filtered through 0.22 μm, and allowed to reach optical density at 600 nm (OD_600nm)_ ≈ 0.8–0.9. Cells were harvested and centrifuged, and the pellets were washed with phosphate buffer solution (PBS, pH 7.4), aliquoted, and frozen. The aliquots were used to determine the concentration of bacterial cells by making serial dilutions of 1:10 in Middlebrook 7H10 agar supplemented with 0.5% glycerol and 10% OADC. Vaccination doses were corroborated with the count of colony-forming units in Middlebrook 7H10 agar culture after 3 weeks of incubation at 37°C.

### 2.3 BCG immunization

BCG at a concentration of 2 × 10^4^ colony-forming units (CFU) in 50 μL of physiological saline solution was subcutaneously administered 60 days prior to infection (day 0) at a single dose in the dorsal area at the level of the scapular region of each mouse using 1-mL syringes and 27 G × 13-mm needles.

### 2.4 *T. cruzi* infection challenge

At day 60 post-immunization, the mice were inoculated with a low inoculum size of 225 blood trypomastigotes (BT) of the Ninoa *T. cruzi* strain (MHOM/MX/1994/Ninoa)—which has been maintained in the laboratory through periodic passages *in vivo* in a murine model—as described below. Blood sampling was carried out on each mouse undergoing the acute state of Chagas disease (21 to 25 days after infection—at parasitemia peak) using a tube rodent holder to safely immobilize the mouse and keep its tail exposed. Vasodilation of the caudal vein was caused by intermittently massaging the tail, and a longitudinal superficial cut was made with a sterile scalpel blade on the dilated vein after asepsis of the area. Gentle pressure was applied, and 10 μL of blood was collected with a micropipette followed by hemostasis using a wound healing dressing. The collected blood was employed to make a 1:50 dilution with saline solution, the parasites were counted in a Neubauer counting chamber, and the volume was adjusted to 200 µL of saline solution to intraperitoneally infect each mouse using 1-mL syringes and 27 G × 13-mm needles.

### 2.5 Parasitemia and survival rate

BT counts were performed using the modified Petana technique (28) every other day from day 10 post-infection and until negative counts were recorded three consecutive times. Briefly, from the caudal vein 10 μL of blood was obtained, which was diluted in 490 μL of saline solution (1:50 dilution). The Neubauer counting chamber was loaded with 10 μL of the dilution, and the BT were quantified in the quadrants used for the leukocyte cell count. Mice survival was recorded daily until the end of the experiment (240 days post-infection—dpi—when euthanasia was performed) to obtain the accumulated mortality rate.

### 2.6 Health condition evaluation

A baseline record of body weight and clinical signs of the animals was made at the beginning of the experiment and throughout the acute and chronic stages of the disease. Weighing of all the mice was carried out with a granataria balance (Sartorius, BL 1500S, Tepotzotlán, Edomex, Mexico). In order to know the health state, a scoring scale was implemented, in which a value was assigned to the visible signs of the clinical condition of the animals as described in **Table 2**.

**Table 2:** Physical condition based on a scoring scale according to the health condition examination.

Scoring scale	Physical state description
1	Without visible signs
2	Mild piloerection
3	Marked piloerection and/or moderate adynamia
4	Hunched spine, marked adynamia, alopecia, and/or abdominal distension
5	Cachexia and/or paralysis of hindquarters

### 2.7 Collection, handling, and storage of blood samples

From the caudal vein, 200–300 μL of blood was collected in 1.5-mL microcentrifuge tubes without an anticoagulant, kept at room temperature until clot retention, and then centrifuged at 3,500 rpm for 15 min at 4°C (Sorvall^®^/DuPont^®^, model RMC-14, Waltham, MA, USA). Aliquots of the sera were made in 0.6-mL microcentrifuge tubes and stored in a freezer at -20°C until use. Samples were collected five times for all groups: (i) prior to the experiments to be used as a reference (pre-immunization), (ii) on day 60 after the immunization and 1 day before infection (post-immunization/pre-infection), (iii) on day 40 after the infection—in the acute phase of the disease (40 dpi), (iv) at 5 months post-infection (5 mpi), and finally (v) at necropsy (8 mpi).

### 2.8 Determination of anti-PPD IgG antibodies

The search for anti-PPD (purified protein derivative, PRONABIVE, obtained from *Mycobacterium bovis* AN5) immunoglobulin G (IgG) antibodies in the sera of mice immunized with *M. bovis* BCG strain was carried out by enzyme-linked immunosorbent assay (ELISA) as described previously (29): 100 ng of bovine PPD in 200 μL of carbonate buffer (NaCO_3_/NaHCO_3_, pH 9.6) was added to each well of Costar 96-well plates (Corning, USA, catalog 2592) and incubated at 37°C for 1 h. Then, the wells were washed five times with 300 μL of 0.05% Tween-20 in PBS (PBS-T), and 200 μL of blocking solution (0.5% bovine serum albumin—BSA—in PBS) was placed for incubation at 37°C for 20 min. The serum samples were diluted in this blocking solution by adding 2 μL of mouse sera (1:100 in PBS) and incubated at 37°C for 1 h. The plates were washed five times with 300 μL of PBS-T. After washing, the peroxidase-labeled rabbit anti-mouse IgG secondary antibody (Novus Biologicals, Centennial, CO, USA) was added at 1:10,000 dilution in PBS-T and incubated for 1 h at room temperature. After washing five times, 150 μL of peroxidase substrate OPD (*orto*-phenylendiamine dihydrochloride, Sigma Aldrich, St. Louis, MO, USA) in citrate buffer at pH 4.5–0.03% H_2_O_2_ was added and incubated at room temperature. At 10 min after adding the substrate, 50 μL of 5 N H_2_SO_4_ was added to stop the reaction, and the OD_450 nm_ was determined using a microplate reader (BioTek 800TS, Winooski, VT, USA). ELISA measurements were performed in duplicate in two independent experiments, the cutoff value was obtained from a negative control serum plus three standard deviations (SD), and a serum from mouse infected with *M. tuberculosis* with 3 months of infection was used as positive control.

### 2.9 Bioinformatic analysis by OrtoVenn 3 program

OrtoVen 3 includes 90 species of protists and can do a comparative analysis of up to 12 species. In the present study, only two species were added for input data: *Leishmania donovani* and *Trypanosoma cruzi* from the built-in database. Data for each species was manually uploaded to the platform in fasta (.fasta) or compressed fasta (.fasta.tar.gz,.fasta.zip) file formats. Subsequently, the analysis function introduction and parameters were selected for the identification and visualization of orthologous clusters. The program was run, and the results were obtained.

### 2.10 Determination of anti-*T. cruzi* IgG antibodies

Evaluation of humoral immune response by the search of anti-*T. cruzi* IgG antibodies in the sera of BCG-immunized/*T. cruzi*-infected and *T. cruzi*-infected mice, as well as IgG1 and IgG2a subclasses for knowing if a Th1, Th2, or a mixed Th1/Th2-type immune response is triggered, was performed by ELISA as described previously (30): 1 μg/mL of a whole protein extract of *T. cruzi* INC-9 isolate in 200 μL of carbonate buffer (NaCO_3_/NaHCO_3_, pH 9.6) was added to each well of Costar 96-well plates (Corning, USA, catalog 2592) and incubated at 37°C for 1 h. The wells were washed five times with 300 μL of 0.05% PBS-T, and 200 μL of BSA was placed for incubation at 37°C for 20 min. Serum samples were diluted in this blocking solution by adding 1 μL of mouse sera (1:200 in PBS) and incubated at 37°C for 1 h. The plates were washed five times with 300 μL of PBS-T. After washing, the peroxidase-labeled rabbit anti-mouse IgG and IgG subclasses (IgG1 and IgG2a) of secondary antibodies (Novus Biologicals, Centennial, CO, USA) were added at 1:10,000 dilution in PBS-T and incubated for 1 h at room temperature. After washing five times, 150 μL of peroxidase substrate OPD in citrate buffer at pH 4.5–0.03% H_2_O_2_ was added and incubated at room temperature. At 10 min after adding the substrate, 50 μL of 5 N H_2_SO_4_ was added to stop the reaction, and the OD_495 nm_ was determined using a microplate reader (BioTek 800TS, Winooski, VT, USA). ELISA measurements were performed in duplicate in two independent experiments, the cutoff value was obtained from a negative control serum plus three SD, and a serum from mouse chronically infected with *T. cruzi* was used as positive control.

### 2.11 Determination of serum cytokines

The cytokines interleukin (IL) IL-1β, IL-2, IL-12, IL-18, interferon (IFN) IFN-γ, tumor necrosis factor (TNF) TNF-α (Th1-type immune response), and IL-4, IL-6, and IL-10 (Th2-type immune response) at 8 mpi (euthanasia time) were measured by using the bead-based immunoassay technique (LEGENDplex^®^ Custom mouse 9-plex Panel, San Diego, CA, USA) according to the manufacturer’s instructions using a flow cytometer (Beckton-Dickinson BD^®^, Aria Fusion, Franklin Lakes, NJ, USA). After acquiring the data from flow cytometer files, they were analyzed using BioLegend’s LEGENDplex™ Data Analysis Software available on biolegend.com/en-us/legendplex, and the cytokine concentrations were calculated from the standard curve.

### 2.12 Anatomopathological description and visceral megas

Euthanasia method was carried out at 8 mpi, and macroscopic findings during the directed necropsy were described. The heart, skeletal muscle, spleen, intestines (ileum and colon sections), popliteal lymph nodes, esophagus, lung, and brain were recovered aseptically.

Prior to euthanasia, the body weight of each mouse was obtained, and the weight of the heart, the spleen, and the popliteal lymph nodes were registered as well during the necropsy procedure. The organ indices were calculated by applying the following formula: organ weight/body weight × 100. Cardiomegaly, splenomegaly, and/or lymphadenopathy were considered when the organ index was significantly higher than that observed in the organs from uninfected control animals (HEALTHY group) (31).

### 2.13 Histopathological evaluation

The recovered organs were immediately washed with isotonic saline solution (0.9% NaCl), collected in a 50-mL conical tube with 10% formaldehyde to fix them at room temperature for at least 2 days, and then processed. Briefly, the organs were cut for their subsequent dehydration and fixation in a sample processor (Leica^®^, model TP1020, Wetzlar, HE, Germany) for 12 h. They were included in paraffin, and 5-µm-thick sections were made with a microtome (Leitz^®^, model 1512, Wetzlar, HE, Germany). The tissue samples were stained with hematoxylin and eosin (H&E), and mount medium for microscopy (Hycel^®^, Zapopan, Jalisco, Mexico) was applied in order to preserve their integrity.

In order to quantify histological damage to the heart and skeletal muscle, inflammation was scored on a scale of 1 to 5, where 1 = no abnormalities, 2 = one focus of inflammatory cells/field, 3 = more than one inflammatory focus/field, 4 = generalized coalescing foci of inflammation or disseminated inflammation with minimal cell necrosis and preserved tissue integrity, and 5 = diffuse inflammation, with severe tissue necrosis, interstitial edema, hemorrhage, and loss of tissue integrity (32). For the remaining organs, pathological anomalies such as hyperplasia, infarct, hemorrhage, etc., were described.

### 2.14 Statistical analysis

The results were analyzed by Kruskal–Wallis test or one-way analysis of variance (ANOVA) and two-way ANOVA tests according to the behavior of the data. *t*-test and Kaplan–Meier test were used to analyze parasitemia and the survival assays. In all cases, GraphPAD PRISM software (version 8) was used, and differences were considered significant when *P* < 0.05.

## 3 Results

### 3.1 Parasitemia and survival rate

To assess the establishment and progression of experimental infection with *T. cruzi*, monitoring of the levels of parasitemia in the mice from the different infected groups was performed.

**Figure 1:**
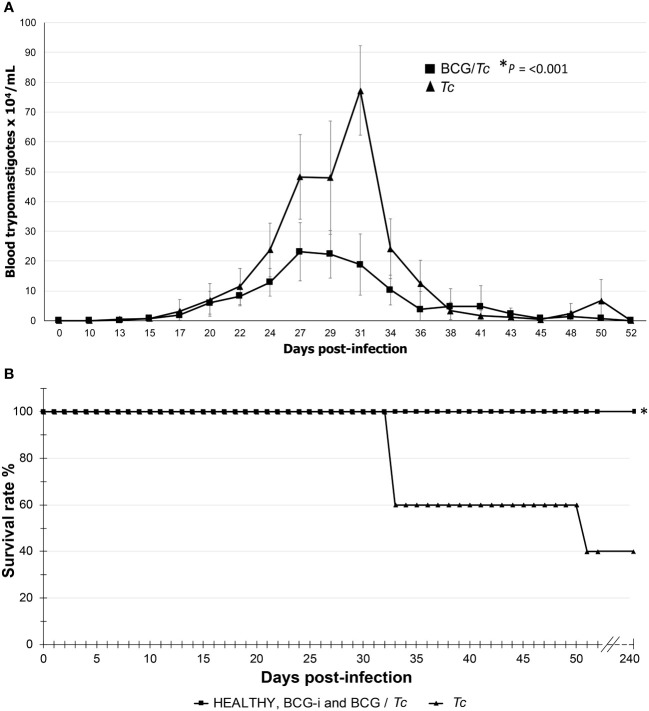
Parasitemia curve **(A)** and survival rate **(B)** of BCG-immunized or nonimmunized mice infected with *T. cruzi*. The values on **(A)** show the mean ± SD of each group and are representative of two independent experiments with equivalent results. At the peak of infection (days 27 to 31), the numbers of parasites were compared by *t*-test, and significant difference was indicated (*) when *P* ≤ 0.05. Values on **(B)** are representative of two independent experiments. Kaplan–Meier curves demonstrating a significant difference when *P ≤ 0.05 between the immunized or unimmunized/infected groups compared to the HEALTHY group.

Parasitemia was detectable from 13 dpi and ended at 52 dpi (the first of three consecutive days when BT counts equal to zero were obtained) for the two infected groups, *Tc* and BCG/*Tc* (**Figure 1A**); these results demonstrated the successful establishment of the infection and the duration of the acute stage.

The parasitemia peaks were the following: for the *Tc* group at 31 dpi with 7.725 **×** 10^5^ parasites/mL of blood and for the BCG/*Tc* group at 27 dpi with 2.313 **×** 10^5^ parasites/mL of blood. This significantly lower parasite burden (*P* = 0.001) suggests that immunization with the BCG strain was beneficial in controlling the acute stage of the infection.

In order to know what the effect of immunization with BCG strain might have on survival, it was monitored that there were no deaths in those animals from the BCG-immunized groups. As expected, immunization alone did not compromise the life of the mice that received the vaccine since the BCG-i group showed 100% survival rate (**Figure 1B**). The BCG/*Tc* group had also 100% survival; this suggested that the BCG immunization conferred a favorable effect since, despite being infected with the parasite, all mice survived, which is consistent with the decrease in parasitemia in this same group.

The *Tc* group presented a survival rate of 40% (**Figure 1B**). The deaths occurred only during the acute stage; during the remaining 6 mpi, there was no change in the survival rates.

### 3.2 Clinical status

#### 3.2.1 Physical condition

All mice were clinically evaluated every third day by physical status examination at the acute stage and at necropsy according to the scoring scale described in **Table 2**. The BCG-i and HEALTHY groups had no change in this parameter throughout the development of the experiment; they started with a score of 1, and until the day of euthanasia (8 months post-infection—mpi) they remained with this same value.

**Figure 2:**
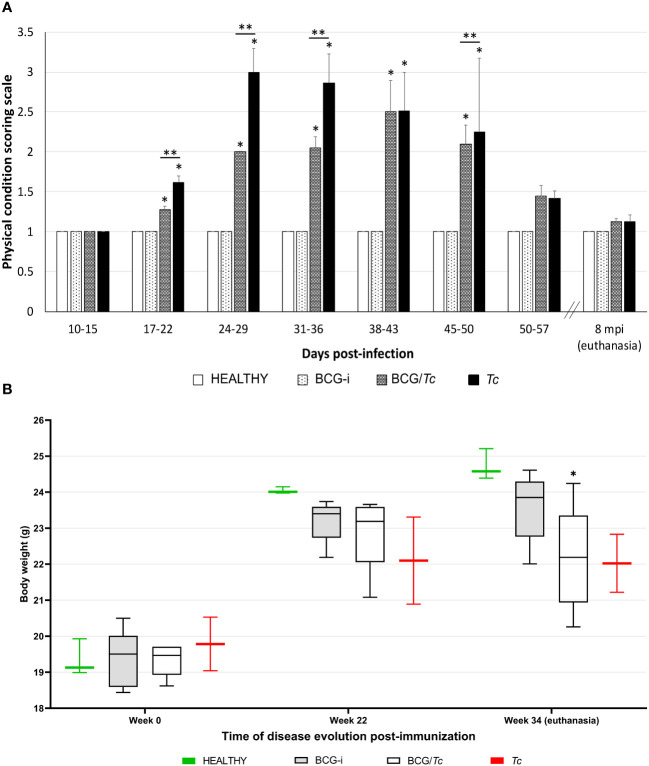
Clinical status **(A)** and body weight **(B)** of BCG-immunized or nonimmunized mice and infected or not with *T. cruzi*. The values show the mean with SD for each group and are representative of two independent experiments with equivalent results. Scoring scale on **(A)** and body weight on **(B)** values from each time were analyzed through multiple comparisons among groups by two-way ANOVA test followed by Dunnett’s as the *post-hoc* test, and significant difference is shown (*) when *P* ≤ 0.05 compared to the HEALTHY group and (**) when compared to BCG/*Tc* and *Tc* groups on **(A)**.

There were visible signs of disease in the infected animals from 20 dpi; those mice from the immunized/infected group (BCG/*Tc*) had a score of 2, while for unvaccinated and infected mice (*Tc*) their score was 3 (**Figure 2A**). From 24 to 29 dpi, the scores from the *Tc* group were increasing by three to four values until the first death was registered on day 34. In this group, the increase in physical condition scores and death was of rapid evolution. In contrast, in mice from the BCG/*Tc* group, scores of 2 were maintained until 38–43 dpi and at the end of the acute stage (45–50 dpi). At the time when parasitemia began to increase, the difference in clinical signs was evident between infected animals that received BCG vaccination and those that were not vaccinated as also demonstrated by the analysis of each group over time (**Figure 2A**); this suggests that BCG significantly decreased the signs of the disease caused by the *T. cruzi* infection. When the parasitemia level was undetectable (52 dpi), the scores in all the surviving mice from any infected group decreased to values of <1.5, which were maintained until euthanasia (8 mpi).

#### 3.2.2 Body weight

The health status of all the infected animals deteriorated over the time of the development of the experiment as demonstrated by the analysis of each group over time, observing a loss of muscle mass in both infected groups BCG/*Tc* and *Tc*; however, only the former had a statistical significance (*P* = 0.0189 vs. *P* = 0.0539, respectively) (**Figure 2B**).

### 3.3 Anti-PPD antibodies

In order to know whether BCG immunization induced an immune response in BALB/c mice, the ability to recognize PPD was evaluated using the ELISA technique. High levels of anti-PPD IgG were obtained in mice from the BCG-i and BCG/*Tc* groups without significant difference at 5 mpi; however, at 8 mpi (euthanasia), the anti-PPD levels increased significantly in the BCG/*Tc* group compared to mice that were only immunized but not infected with *T. cruzi* (**Supplementary Figure 1**).

With the aim of knowing if mouse sera could be reacting nonspecifically against PPD, other antigen extracts from Gram-negative and Gram-positive bacteria and two protozoans were tested for ELISA. The sera from mice immunized with BCG or immunized and infected with *T. cruzi* did not react with either of the two bacterial genera—*Escherichia coli* and *Lactobacillus* spp.—nor with the protozoan *Entamoeba histolytica*; however, in some sera from the *Tc* and BCG/*Tc* groups, there was cross-reactivity with *Leishmania donovani*, which is a protozoan member of the Trypanosomatidae family like *T. cruzi*, but not with sera from the BCG-i group (**Supplementary Figure 2**).

To support this result, a bioinformatic analysis was carried out in OrtoVenn 3 program with the purpose of searching conserved protein sequences in *T. cruzi* and *L. donovani*; the analysis demonstrated the presence of 5,951 orthologous genes (**Supplementary Figure 3**). More than 70% of the *T. cruzi* proteins have homology with proteins and/or polypeptides of *Leishmania donovani*, and of 100% of the *L. donovani* proteins, 98.28% are homologous to *T. cruzi.*

### 3.4 Anti-*T. cruzi* antibodies

#### 3.4.1 Total IgG-anti-*T. cruzi*

**Figure 3:**
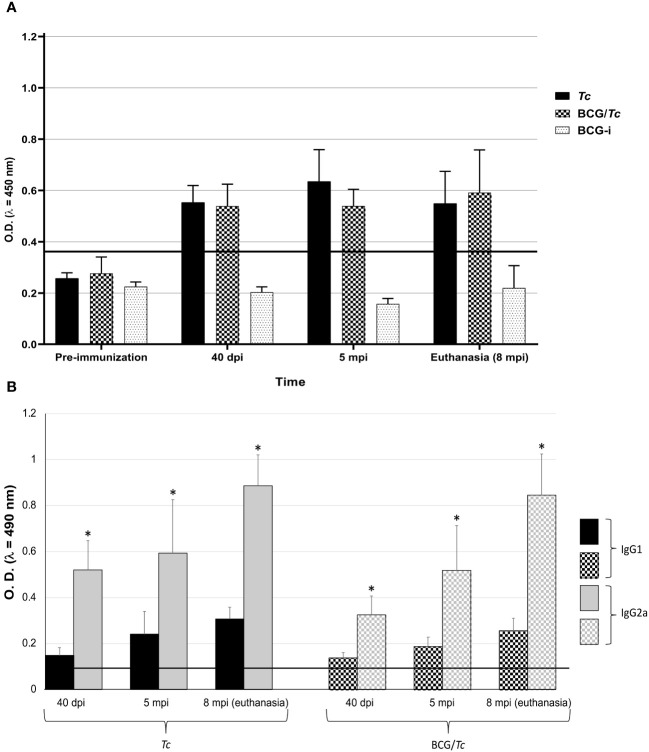
Total IgG **(A)**, and IgG1 and IgG2a **(B)** anti-*T. cruzi* from BCG-immunized or nonimmunized mice and infected or not with *T. cruzi*. Values of each group are representative of two independent experiments with equivalent results. Data are presented as mean with S.D. from individual O.D._490nm_ values from each group at each time. A One-Way ANOVA test was used for statistical analysis and significant difference was indicated (*) when P ≤ 0.05. Black line shows the cut-off value.

The *Tc* and BCG/*Tc* groups presented reactivity against *T. cruzi* with a similar behavior, which was observed in an ascending manner throughout the time of infection without showing statistically significant differences (**Figure 3A**).

#### 3.4.2 IgG1 and IgG2a anti-*T. cruzi*

In order to know whether a Th1, Th2, or a mixed Th1/Th2-type immune response is triggered after immunization with BCG and infection with *T. cruzi*, the IgG subclass levels were determined, which have different effector functions depending on their predominance. In both infected groups (*Tc* and BCG/*Tc*), the levels of the IgG2a subclass were significantly higher than those of the IgG1 subclass at all times of *T. cruzi* infection, with a mean IgG2a/IgG1 ratio of almost three times (2.95 and 2.81 for *Tc* and BCG/*Tc* groups, respectively) (**Figure 3B**); these results indicated a polarized immune response that suggests protective immunity against infection is associated. BCG immunization had no different effect than infection with *T. cruzi* alone on the production of antibodies of either subclass throughout the entire experiment.

### 3.5 Determination of serum cytokines

**Figure 4:**
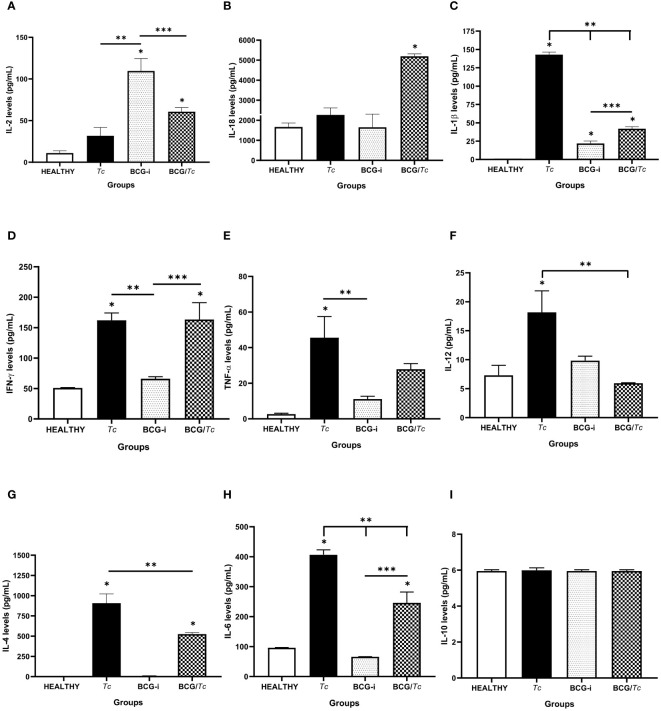
Serum cytokine levels in BCG-immunized or nonimmunized mice and infected or not with *T. cruzi*. **(A)** IL-2, **(B)** IL-18, **(C)** IL-1β, **(D)** IFN-γ, **(E)** TNF-α, **(F)** IL-12, **(G)** IL-4, **(H)** IL-6, and **(I)** IL-10 were detected at picogram per milliliter. The levels of each cytokine were analyzed through multiple comparisons among groups by one-way ANOVA test followed by Tukey’s as the *post-hoc* test, and significant difference is shown (*) when *P* ≤ 0.05 compared to the HEALTHY group, (**) when compared to the *Tc* group, and (***) when compared to the BCG-i group.

The production of nine cytokines was evaluated in serum samples obtained at euthanasia time (8 mpi), with six belonging to Th1-type response (IL-2, IL-18, IL-1β, IFN-γ, TNF-α, and IL-12) and three to Th2 (IL-4, IL- 6, and IL-10) (**Figure 4**).

Chronic infection (8 mpi) with *T. cruzi* induced the production of significantly elevated levels of IL-1β, IFN-γ, TNF-α, and IL-12; however, IL-2 and IL-18 had similar levels to those in the HEALTHY group. This same group of mice (*Tc*) also had significantly high levels of IL-4 and IL-6, so it can be suggested that at 8 mpi a predominance of Th1-type response is occurring.

In the case of the BCG/*Tc* group, cytokine production was balanced since TNF-α and IL-12 were not induced, and the levels of IL1-β were significantly low compared to the *Tc* group, which did not receive BCG immunization. On the other hand, BCG administration alone only had a positive effect on the production of IL-2 and IL-1β.

### 3.6 Macroscopic findings at the chronic phase

#### 3.6.1 Examination of necropsy organs

Euthanasia was performed at 8 mpi, and the findings observed during necropsy were registered. In a mouse from the BCG-i group (1/10, 10%), an alopecic area was found on the dorsal region (**Supplementary Figure 4A**), most likely as a consequence of subcutaneous immunization at that site. In two mice from the *Tc* group (2/10, 20%), a white/yellowish area was found on the surface of the heart (**Supplementary Figure 4B**), corresponding to calcific pericarditis when histological examination was performed.

#### 3.6.2 Cardiomegaly, splenomegaly, and lymphadenopathy

Megalias are part of the macroscopic findings as well as a sign of chronic ChD; to evaluate the effect of immunization with BCG strain on this manifestation in the experimental groups, the cardiac, splenic, and lymph node indices were calculated. To consider megalia, there must be a statistically significant increase in organ size when compared to the HEALTHY group.

**Figure 5:**
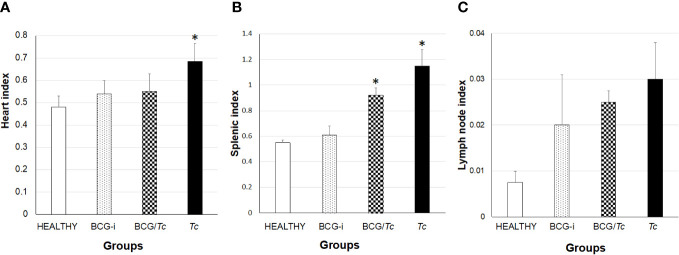
Heart **(A)**, splenic **(B)**, and lymph node **(C)** indices in BCG-immunized and nonimmunized mice and infected or not with *T. cruzi*. The values show the mean of the indices ± SD of each group and are representative of two independent experiments. The mean of the indices was compared with a Kruskal–Wallis test, and it was considered that there is statistical significance when (*) *P* ≤ 0.05 upon comparing all the groups against the HEALTHY group.

Cardiomegaly was detected only in the *Tc* group (**Figure 5A**), which was expected since *T. cruzi* has a tropism toward the heart and is common in ChD. Of the infected groups, the BCG-immunized mice (BCG/*Tc*) presented lower values than those infected with *T. cruzi* (*Tc* group), which could be indicative of possible protection by BCG against heart disease related to *T. cruzi* infection. On the other hand, the BCG-i group did not show any effect related to the immunization on heart enlargement.

Both infected groups, BCG/*Tc* and *Tc*, presented splenomegaly (**Figure 5B**), indicating that even in the chronic stage the immune system remains active both with the BCG stimulus coupled with the infection and with the infection alone.

In the immunized and immunized/infected groups, there was a clear increase in lymph node indices, but no group had lymphadenopathy (**Figure 5C**).

### 3.7 Microscopic findings at the chronic phase

#### 3.7.1 Histopathology description

**Figure 6:**
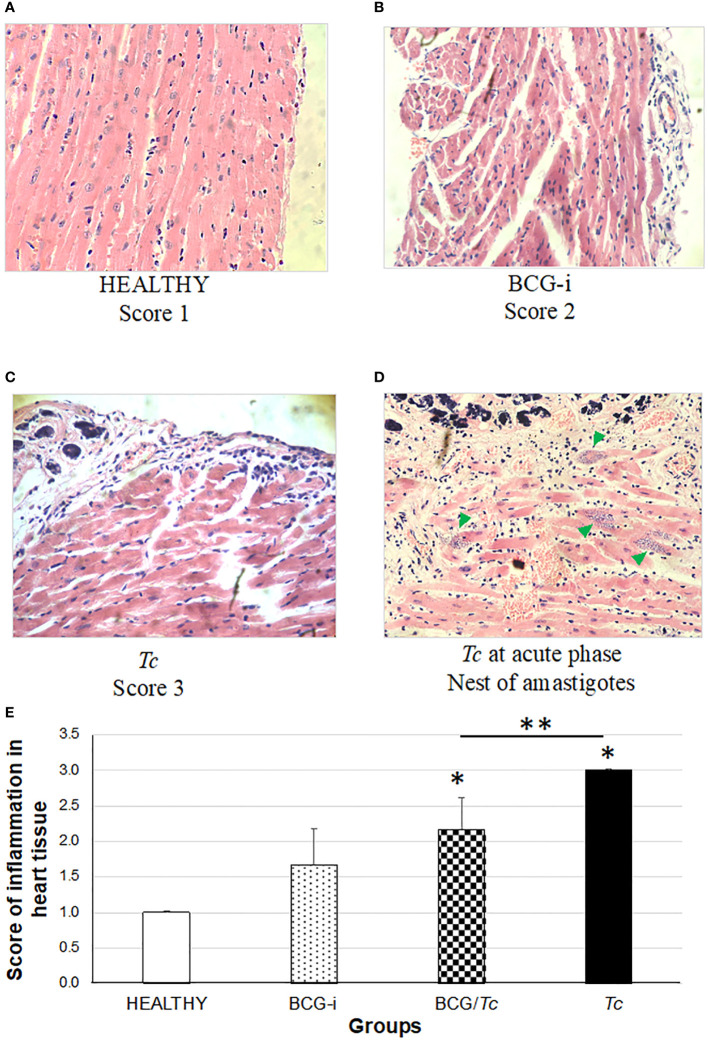
Degree of damage and inflammation of heart **(A–D)** at the chronic phase of ChD. **(A)** Representative microphotograph of score 1: no alterations (corresponding to a mouse tissue from the HEALTHY group), **(B)** score 2: one focus of inflammatory cells per field (corresponding to a mouse tissue from the BCG-i group), **(C)** score 3: more than one or a few inflammatory cell foci per field (corresponding to a mouse tissue from the *Tc* group), and **(D)** nests of amastigotes (green arrows) corresponding to a mouse tissue from the *Tc* group at the acute phase of ChD. The values on **(E)** show the mean of the inflammation scores in heart tissue ± SD of each group and are representative of two independent experiments. The mean of the scores was compared using a one-way ANOVA test; it was considered that there is statistical significance when *P* ≤ 0.05 upon comparing each group against the HEALTHY (*) and against the *Tc* groups (**) at *P* ≤ 0.05.

**Figure 7:**
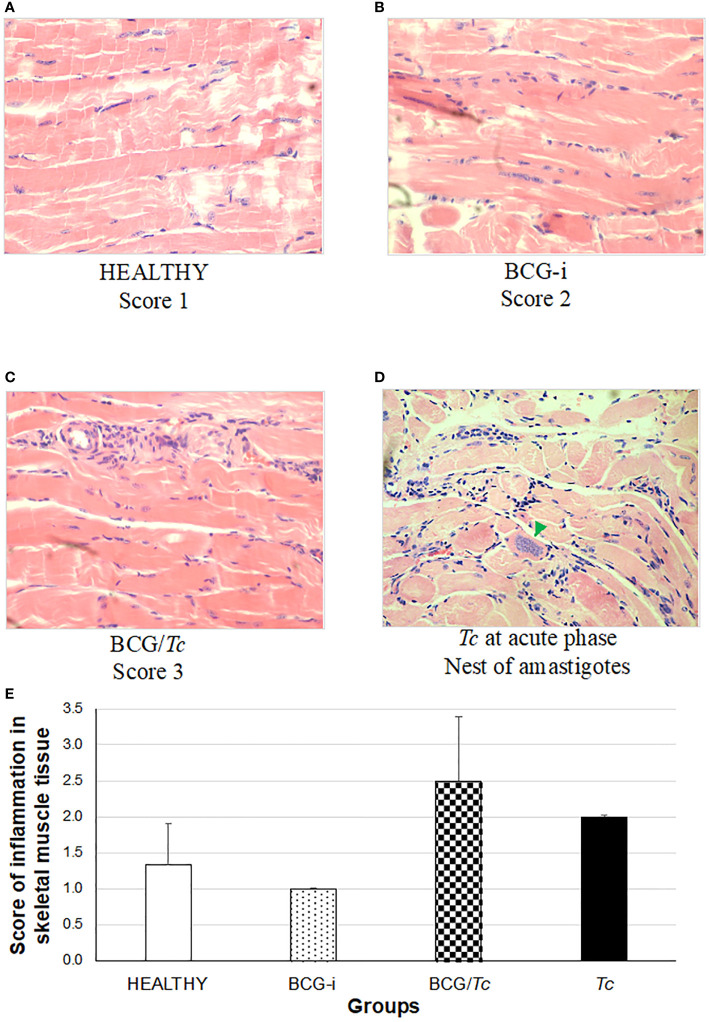
Degree of damage and inflammation of skeletal muscle **(A–D)** at the chronic phase of ChD. **(A)** Representative microphotograph of score 1: no alterations (corresponding to a mouse tissue from the HEALTHY group), **(B)** score 2: one focus of inflammatory cells per field (corresponding to a mouse tissue from the BCG-i group), **(C)** score 3: more than one or a few inflammatory cell foci per field (corresponding to a mouse tissue from the BCG/*Tc* group), and **(D)** nest of amastigotes (green arrow) corresponding to a mouse tissue from the *Tc* group at the acute phase of ChD. The values **(E)** show the mean of the inflammation scores in skeletal muscle tissue ± SD of each group and are representative of two independent experiments. There were no statistically significant differences when the values were analyzed using a one-way ANOVA test upon comparing each group against the HEALTHY and against the *Tc* groups (*P* ≤ 0.05).

The presence of tissue alterations, mainly in the heart (**Figures 6A–C**) and skeletal muscle (**Figures 7A–C**), is one of the common manifestations of *T. cruzi* infection. Inflammation in the heart manifested mainly as pericarditis, although in some isolated cases myocarditis also occurred with very low levels.

#### 3.7.2 Inflammation severity

In this study, a rating scale for the degree of tissue injury in these organs was used to quantify histological damage by observing lymphocytic and mast cell infiltrates and tissue integrity. Since the tissues were obtained during the chronic stage, the presence of amastigotes’ nests (characteristic of the acute stage) was not observed in any case. However, in those animals in the *Tc* group that died in the acute phase and whose organs could be obtained, nests of amastigotes were observed (**Figures 6D**, **7D**).

The heart inflammation score was considerably high in mice from the *Tc* and BCG/*Tc* groups with statistical significance compared to the HEALTHY group; although the BCG-i group showed inflammation levels greater than 1, with minimal alteration in the heart, the statistical analysis revealed no differences with respect to the HEALTHY group (**Figure 6E**). Although the BCG/*Tc* group presented high inflammation scores, these were significantly lower than those presented by the *Tc* group, suggesting that the BCG immunization ameliorated the inflammation caused by *T. cruzi*.

In inflammation in skeletal muscle (**Figure 7E**), no statistical significance was found between the groups; however, the highest degree of inflammation was observed in *T. cruzi*-infected mice with or without immunization, i.e., *Tc* and BCG/*Tc* groups.

## 4 Discussion

The use of BCG as a vaccination strategy against experimental ChD has already been proposed; for example, Kuhn and collaborators (21) found that immunization with BCG had no effect on the development of parasitemia or longevity in *T. cruzi*-infected C3H(He) mice. However, there were some differences in the distribution of parasites in the organs between the mice immunized with BCG and the control mice. On the other hand, it was established that vaccination with BCG plus dead *Leishmania* promastigotes reduced the acute infection by *T. cruzi* in a murine model, increasing survival time and decreasing parasitemia and mortality (33). The results of parasitemia in this study are comparable with previous ones (30, 34), in which two peaks of parasitemia were described with magnitudes of 10^6^ parasites/mL of blood in a similar infection with the Ninoa strain intraperitoneally with 150 BT. Other research groups (35, 36) have reported peaks of parasitemia that reach levels of 10^6^ parasites/mL of blood in BALB/c mice infected with two Mexican strains Querétaro and Ninoa belonging to DTU TcI, in which the infection inoculum was around 10^4^ BT.

Previously, survival rates of around 80% have been reported for mice infected with the same Ninoa *T. cruzi* strain. These percentages are high compared to these from the present study (40%), which suggests that by maintaining the strain *in vivo* its virulence is enhanced since the infection becomes more aggressive, causing greater mortality during the acute stage (30, 34).

Vaccination with BCG plus dead *Leishmania* promastigotes and a subsequent infection with *T. cruzi* carried out by Araujo and colleagues (1999) (33) significantly prolonged the mean survival times in mice vaccinated with BCG-*Leishmania* compared to the groups that received PBS, BCG, or *Leishmania* alone, results which are similar to those found in the present investigation with 100% of survival rate in those mice from the BCG/*Tc* group. Therefore, BCG vaccine used as an immunostimulant of the response against a different microorganism, *T. cruzi*, was beneficial by preventing mortality.

The evaluation of physical condition parameters and body weight recording in Chagas disease is not frequently performed; therefore, information is scarce. Although a scale was not used to assess the clinical status of the animals, the physical condition of mice infected with the Querétaro strain of *T. cruzi* was described (36). These authors reported bristly hair on the back, continuous tremor throughout the body, and loss of mobility of the hind limbs at around 13–15 dpi. Such manifestations coincided with the increase in the parasite load in the blood, and they recovered some mobility of the hind limbs at 90 dpi. In the present work, the highest scores were at 24–29 dpi in both infected groups, which, as in the aforementioned study, coincide with the peaks of parasitemia (27, 29, and 31 dpi). The BCG/Tc group showed lower scores indicating a protective effect of immunization, suggesting that BCG had an immunostimulatory role.

BCG immunization induced a specific humoral immune response; possibly at the time of infection, in addition to resulting in the production of specific antibodies against the parasite, there was probably also a clonal expansion of the memory cells produced in the vaccination. The immunological response toward *M. bovis* BCG was dominant over the response against *T. cruzi*, presumably because bacterial lipoproteins—which are often among the most immunogenic bacterial antigens (14)—were immunodominant over parasitic antigens. It has been described that most of the structural components of the mycobacterial envelope are lipids associated with carbohydrates (glycolipids), glycosylated phospholipids, or complex carbohydrates substituted with mycolic acid or peptides. The glycosylated portions of these molecules are recognized by various receptors in macrophages and other cell types, such as T lymphocytes, which is why they are important in the interaction with the components of the innate and host-specific immune response (37).

In this work, BCG had beneficial effects in mice infected with *T. cruzi* since a significant decrease in parasite load and greater survival were observed. Regarding cellular immunity, a differential response was also observed in the production of cytokines between the group infected with the parasite and that was previously BCG-vaccinated (BCG/*Tc* group) compared to that which did not receive the vaccination (*Tc* group). Vaccination improved the control of *T. cruzi* infection probably due to the stimulation of a specific T cell proliferative response and the production of IFN-γ. In the infected mice, there was an inflammatory response, while in those with trained immunity the response was balanced, resulting in a better prognosis of the disease in the chronic stage. These results agree with those reported by others who obtained levels of IFN-γ, IL-12, and IL-10 that were different from those of the non-immunized groups by immunizing mice with a combination of BCG and dead *Leishmania* promastigotes and then infecting them with *T. cruzi* (27). It was explored that this trained immunity strategy in human monocytes, and it was reported that BCG vaccination protected against experimental viral infection of yellow fever virus on the basis that the reduction in viremia was directly related to the positive regulation of IL-1β (25). García-Hernández et al. (2009) argue that the antitumor effects of BCG in bladder cancer seem to be related to a transient increase in several cytokines and the presence of immunocompetent leukocytes (38). Mechanisms have previously been described to explain BCG-trained immunity, including epigenetic reprogramming, for example, based on methylation and acetylation patterns; metabolic reprogramming in which an increase in the promoters of genes that encode enzymes has been observed, such as those involved in metabolic pathways such as cell activation and proliferation; and, finally, long-term protection mediated by the change in the bone marrow microenvironment that influences the maturation of cells of the immune system and the consequent cytokine production both in the initial and in the chronic stages (39).

The most common macroscopic finding in the *Tc* group was a white area on the surface of the heart, which is associated with calcifying pericarditis. Constrictive or calcifying pericarditis is a pathology that is characterized by the compression of the heart by a thickened and rigid pericardium that makes ventricular diastolic filling difficult, and it is a rare clinical entity that may constitute the final evolutionary stage of many genetic inflammatory, traumatic, and infectious processes, among others. This condition has been previously described in the case of a 41-year-old woman with constrictive pericarditis associated with Chagas cardiomyopathy (40).

Visceral megas have been previously evaluated in the murine and canine model of ChD when evaluating the therapeutic effect of two therapeutic compounds, reporting the absence of cardiomegaly in animals infected with *T. cruzi* and treated with the different products (34, 41). In the present work, BCG vaccination was able to prevent cardiomegaly. Cardiac involvement is the most serious manifestation of ChD in the chronic phase, and several theories have been proposed about this pathogenesis (42).

Splenomegaly is a sign related to the prominent proliferation of lymphoid cells characteristic of the polyclonal activation of B and T cells (43), which is an immunological response in the chronic stage that was not prevented or potentiated by BCG vaccination in the present study. Splenomegaly and lesions in the spleen due to infection with *T. cruzi* have likewise already been previously reported in a murine model and have been attributed to the activation of macrophages, NK cells, and CD8+ lymphocytes, which leads to an increase in the levels of cytokines with a pro-inflammatory Th1 profile and, at the same time, produces an uncontrolled inflammatory reaction (34, 35).

By comparing two mouse strains with different levels of susceptibility to infection with *T. cruzi*, it was observed that splenomegaly in the acute phase is due to an active immune response that leads to reactive hyperplasia with an increase in the number of lymphocytes and macrophages that culminates in the disintegration of the parasite and necrosis of the parasitized cells (43). In the present study, the determination of splenomegaly and cytokines was performed in the chronic stage of the infection, which showed a directly proportional relationship of both parameters in the BCG/*Tc* and *Tc* groups, which agrees with what was reported by those authors who concluded that TNF-α, probably synthesized by macrophages, was strongly expressed in parasitized sites. Therefore, this cytokine seems to play a primary role in the splenic necrotic changes associated with severe acute infection.

Lymphadenopathy is a manifestation that has been described in the acute stage of ChD (44, 45). However, in 2020, this finding was also described in the chronic stage in a 65-year-old man who received a heart transplant and who, 3 years later, developed inguinal lymphadenopathy, which was due to *T. cruzi* infection as diagnosed by molecular and immunological tests (46). The results in the present study, in which there was no significant lymphadenopathy in the chronic stage, disagree with these observations but are comparable to the work of others (31), in which in the canine model in the chronic stage all animals infected with *T. cruzi* showed cardiomegaly, splenomegaly, and absence of lymphadenopathy.

Regarding the microscopic findings, the results obtained in the present study agree with those reported by other authors, who argue that the factors that contribute and exacerbate the inflammatory response are the local production of cytokines, chemokines, and their receptors; positive regulation of adhesion molecules; complement activation; platelet aggregation and adhesion; and the production and opsonization of antibodies. During the chronic stage, only focal areas of inflammation are generally found in chagasic hearts. Inflammatory cell infiltrates are composed primarily of T cells and macrophages with some eosinophils, plasma cells, and mast cells. In the chronic stage, the presence of intramyocardial parasites induces a latent and continuous inflammatory reaction (47). Myocarditis with the presence of mainly mononuclear cells, CD4 T lymphocytes, CD8 T lymphocytes, and macrophages in mice with chronic infection had been observed (48). Cellular inflammatory infiltrates (87%), degeneration of cardiac fibers (7%), pericarditis (6%), and nests of *T. cruzi* amastigotes were observed in samples from rodents captured in southeastern Mexican rural and suburban areas (49, 50).

Severe inflammation has been considered an important feature of tissue calcification in some clinical conditions. Two mechanisms of calcification are recognized: metastatic calcification (elevated serum calcium and/or phosphate levels resulting in systemic mineralization) and dystrophic calcification (associated with injury, infection, or rheumatic diseases with normal calcium/phosphate homeostasis). Dystrophic calcification is also related to cell death, leading to the release of catabolic enzymes and calcium. Tissue mineralization near inflammatory infiltrates has been observed in cases of aortic calcification, and degradation products of apoptosis may promote this. Furthermore, some studies on experimental ChD have demonstrated an association of TNF-α with vascular calcification; this cytokine is present in both acute and chronic stage inflammation, and in chronic cases it can induce the activation of apoptotic pathways (36). The calcification that was described in the present study is dystrophic.

It was already mentioned previously that there is no literature regarding the use of the BCG strain as an immunomodulator in *T. cruzi* infection. However, the work of others (51) addressed the use of Actinomycetales suspensions on ChD and also evaluated the levels of inflammation in the heart in the chronic stage using the following scale: (1) small foci: slight inflammatory infiltrate with damage to two to three myocardial fibers, (2) medium foci: aggregated infiltrates that involve four to 10 muscle fibers, and (3) large foci: large accumulation of lymphocytes and macrophages with destruction of more than 10 muscle fibers. The number of lesions observed in rats treated with the Actinomycetales suspension was a little higher than in the control group, but less than those observed in the infected group, which is similar to the results obtained in the present study in which BCG vaccination induced inflammation with scores higher than those of the HEALTHY group but lower than those of the *Tc* one. These similar results in mice from the BCG/*Tc* group suggested a possible consequence of better long-term immunoregulation during acute infection since the more effective elimination of parasites would have served to reduce the inflammatory response in the chronic stage, favoring a lower influx of potentially aggressive immunocompetent cells to the cardiac tissue. Furthermore, the lipids and sugars in the cell wall structure of *M. bovis*, as well as Actinomycetales, probably have immunomodulatory effects that affected both innate and adaptive immunity (51).

Inflammatory infiltrates develop mainly in chronic infection and generally appear as a consequence of the formation of nests with amastigotes (52). Several factors have been determined as triggers for the formation of cellular inflammatory infiltrates in individuals with chronic infections, among which are the pathogenesis of the infectious *T. cruzi* genotype, the time of the disease evolution course, and the presence of concomitant infection with other parasites (polyparasitism) as well as the interaction of the parasite genome with that from the infected host and other factors such as age, sex, and the presence of previous infections in the host (50).

Inflammation degree in mice infected with the Ninoa and Querétaro strains in both the heart and skeletal muscle with a scale in which 1 was equivalent to scant inflammation and 2 to diffuse infiltrate was evaluated (53), and inflammation scores in the heart of less than 1 and skeletal muscle scores of 1.5 to 2 in the chronic stage at 90 dpi were reported. These findings do not agree with those obtained in the cardiac tissue of the *Tc* group of the present study since the infection with the Ninoa strain yielded scores of 3; however, immunization with BCG reduced the degree of inflammation in the chronic stage, significantly reducing the score.

This study has some limitations that should be noted. The Ninoa strain used here has been becoming increasingly virulent as it is maintained within an animal model, and the mortality of infected mice might reduce the number of animals in both the acute and chronic stages, therefore potentially affecting the sample size that is used for comparison among experimental groups. The evaluation of the health condition by two parameters—a score according to the observer’s criteria, which must always be done by the same person, and the weight of the animals on a conventional scale—can limit the precision of this measurement. The study would be enriched in this aspect by including more parameters such as temperature measurement, electrocardiographic and echocardiographic records, etc. The determination of serum cytokines only at the end of the study and using a kit of only nine cytokines limit the interpretation of immune response stimulation since the Th17 type or the comparison of a possible differential production over time is not included.

In conclusion, the use of the *Mycobacterium bovis* BCG strain had an immunomodulatory effect by reducing the pathology of *Trypanosoma cruzi* infection during the acute stage by showing a significant reduction in parasitemia, a higher percentage of survival, and a better clinical status of the experimental animals. In addition, there was no differential production of sera antibodies against *Trypanosoma cruzi* in mice that received the BCG vaccine compared with those not vaccinated, but there was a dominant response against PPD. The profile of the cellular immune response demonstrated that the nonspecific immunity in mice infected with *T. cruzi* and previously vaccinated with BCG induced a balanced production of Th1- and Th2-type cytokines, resulting in a better prognosis of the disease in the chronic stage.

## Data availability statement

The original contributions presented in the study are included in the article/**Supplementary Material**. Further inquiries can be directed to the corresponding authors.

## Ethics statement

The animal study was approved by Internal Committee for the Care and Use of Laboratory Animals (CICUAL, for its acronym in Spanish) with registration number: INC/CICUAL/011/2021, approved on 23 Aug 2021. The study was conducted in accordance with the local legislation and institutional requirements.

## Author contributions

MA: Conceptualization, Formal analysis, Funding acquisition, Methodology, Resources, Supervision, Writing – original draft, Writing – review & editing. DM: Methodology, Resources, Writing – original draft, Writing – review & editing. AA: Methodology, Writing – original draft, Writing – review & editing. JR: Funding acquisition, Resources, Writing – original draft, Writing – review & editing. MF: Conceptualization, Formal analysis, Funding acquisition, Resources, Writing – original draft, Writing – review & editing. OR: Conceptualization, Formal analysis, Funding acquisition, Methodology, Resources, Supervision, Writing – original draft, Writing – review & editing.

## References

[1] Echeverria LE Morillo CA. American trypanosomiasis (Chagas disease). Infect Dis Clin North Am. (2019) 33:119–34. doi: 10.1016/j.idc.2018.10.015 30712757

[2] PAHO. Factsheet: Chagas Disease in the Americas for Public Health Workers (2022). Available online at: https://www.paho.org/en/documents/factsheet-chagas-disease-americas-public-health-workers.

[3] Cruz-Alegría I Gutiérrez-Ruiz J Cortés-Ovando D Santos-Hernández N Ruiz-Castillejos C Gómez-Cruz A. Prevalencia y conocimientos de la enfermedad de Chagas en dos comunidades del sureste de México. Rev BioMed. (2021) 32:106–12. doi: 10.32776/revbiomed.v32i2.890

[4] Pérez-Molina JA Molina I. Chagas disease. Lancet. (2018) 391:82–94. doi: 10.1016/S0140-6736(17)31612-4 28673423

[5] Salazar-Schettino PM Bucio-Torres MI Cabrera-Bravo M de Alba-Alvarado MC Castillo-Saldaña DR Zenteno-Galindo EA. Enfermedad de Chagas en México. Rev Fac Med (Méx). (2016) 59:6–16.

[6] Nunes MCP Beaton A Acquatella H Bern C Bolger AF Echeverría LE. Chagas cardiomyopathy: an update of current clinical knowledge and management: a scientific statement from the American Heart Association. Circulation. (2018) 138:e169–209. doi: 10.1161/CIR.0000000000000599 30354432

[7] Bivona AE Alberti AS Cerny N Trinitario SN Malchiodi EL. Chagas disease vaccine design: the search for an efficient *Trypanosoma cruzi* immune-mediated control. Biochim Biophys Acta Mol Basis Dis. (2020) 1866:165658. doi: 10.1016/j.bbadis.2019.165658 31904415

[8] Flores-Valdez MA. After 100 years of BCG immunization against tuberculosis, what is new and still outstanding for this vaccine? Vaccines. (2022) 10:57. doi: 10.3390/vaccines10010057 PMC8778337 35062718

[9] Trunk G Davidović M Bohlius J. Non-specific effects of Bacillus calmette-guérin: A systematic review and meta-analysis of randomized controlled trials. Vaccines (Basel). (2023) 11:121. doi: 10.3390/vaccines11010121 36679966 PMC9866113

[10] Tran V Liu J Behr MA. BCG vaccines. Microbiol Spectr. (2014) 2:MGM2–2013. doi: 10.1128/microbiolspec.MGM2-0028-2013 26082111

[11] Covián C Fernández-Fierro A Retamal-Díaz A Díaz FE Vasquez AE Lay MK. BCG-induced cross-protection and development of trained immunity: implication for vaccine design. Front Immunol. (2019) 10:2806. doi: 10.3389/fimmu.2019.02806 31849980 PMC6896902

[12] Dos Santos JC Vilela Teodoro Silva M Ribeiro-Dias F Joosten L. Non-specific effects of BCG in protozoal infections: tegumentary leishmaniasis and malaria. Clin Microbiol Infect. (2019) 25:1479–83. doi: 10.1016/j.cmi.2019.06.002 31212075

[13] Singh AK Netea MG Bishai WR. BCG turns 100: its nontraditional uses against viruses, cancer, and immunologic diseases. J Clin Invest. (2021) 131:e148291. doi: 10.1172/JCI148291 34060492 PMC8159679

[14] Stover CK Bansal GP Hanson MS Burlein JE Palaszynski SR Young JF. Protective immunity elicited by recombinant bacille Calmette-Guerin (BCG) expressing outer surface protein A (OspA) lipoprotein: a candidate Lyme disease vaccine. J Exp Med. (1993) 178:197–209. doi: 10.1084/jem.178.1.197 8315378 PMC2191093

[15] Langermann S Palaszynski S Sadziene A Stover CK Koenig S. Systemic and mucosal immunity induced by BCG vector expressing outer-surface protein A of *Borrelia burgdorferi* . Nature. (1994) 372:552–55. doi: 10.1038/372552a0 7990928

[16] Edelman R Palmer K Russ KG Secrest HP Becker JA Bodison SA. Safety and immunogenicity of recombinant Bacille Calmette-Guérin (rBCG) expressing *Borrelia burgdorferi* outer surface protein A (OspA) lipoprotein in adult volunteers: a candidate Lyme disease vaccine. Vaccine. (1999) 17:904–14. doi: 10.1016/S0264-410X(98)00276-X 10067697

[17] Mustafa AS. BCG as a vector for novel recombinant vaccines against infectious diseases and cancers. Vaccines. (2020) 8:736. doi: 10.3390/vaccines8040736 33291702 PMC7761935

[18] Bertelli MS Alcantara A Brener Z. BCG-induced resistance in *Trypanosoma cruzi* experimental infections. Tropenmed Parasitol. (1981) 32:93–6. 6789512

[19] Ortiz-Ortiz L Gonzalez-Mendoza A Lamoyi E. A vaccination procedure against *Trypanosoma cruzi* infection in mice by nonspecific immunization. J Immunol. (1975) 114:1424–25. doi: 10.4049/jimmunol.114.4.1424 804010

[20] Hoff R. Killing in *vitro* of *Trypanosoma cruzi* by macrophages from mice immunized with T. cruzi or BCG, and absence of cross-immunity on challege in *vivo* . J Exp Med. (1975) 142:299–311. doi: 10.1084/jem.142.2.299 806649 PMC2189902

[21] Kuhn RE Vaughn RT Herbst GA. The effect of BCG on the course of experimental Chagas' disease in mice. Int J Parasitol. (1975) 5:557–60. doi: 10.1016/0020-7519(75)90049-1 808508

[22] Burgess DE Hanson WL. Heterologous and specific immunization of mice against *Trypanosoma cruzi* . J Parasitol. (1980) 66:340–42. doi: 10.2307/3280831 6771372

[23] Vicco MH Bontempi IA Rodeles L Yodice A Marcipar IS Bottasso O. Decreased level of antibodies and cardiac involvement in patients with chronic Chagas heart disease vaccinated with BCG. Med Microbiol Immunol. (2014) 203:133–39. doi: 10.1007/s00430-013-0326-x 24374613

[24] Peverengo L Prochetto E Rodeles L Valenzuela I Marcipar IS Bottasso O. Antibody profiles induced by Trypanosoma cruzi in chagasic patients with previous or current exposure to mycobacteria. Pathog Dis. (2016) 74:ftw109. doi: 10.1093/femspd/ftw109 27815312

[25] Arts RJW Moorlag SJCFM Novakovic B Li Y Wang SY Oosting M. BCG Vaccination Protects against Experimental Viral Infection in Humans through the Induction of Cytokines Associated with Trained Immunity. Cell Host Microbe. (2018) 23:89–100.e5. doi: 10.1016/j.chom.2017.12.010 29324233

[26] Walk J de Bree LCJ Graumans W Stoter R van Gemert GJ van de Vegte-Bolmer M. Outcomes of controlled human malaria infection after BCG vaccination. Nat Commun. (2019) 10:874. doi: 10.1038/s41467-019-08659-3 30787276 PMC6382772

[27] Araujo Z Heremans H Stordeur P Wissing M Goldman M Castes M. IFN-gamma, IL-4, IL-10 and IL-12 gene expression in BCG-Leishmania vaccination of *Trypanosoma cruzi*-infected mice. Vaccine. (2000) 18:1822–29. doi: 10.1016/s0264-410x(99)00426-0 10699330

[28] Urribarrí RS. Estudio comparativo de dos métodos para valoración cuantitativa de la parasitemia por tripanosomas. KASMERA. (1974) 5:103–10.

[29] Aceves-Sánchez MJ Flores-Valdez MA Pedroza-Roldán C Creissen E Izzo L Silva-Angulo F. Vaccination with BCGΔBCG1419c protects against pulmonary and extrapulmonary TB and is safer than BCG. Sci Rep. (2021) 11:12417. doi: 10.1038/s41598-021-91993-8 34127755 PMC8203684

[30] Rodríguez-Morales O Cabrera-Mata JJ Carrillo-Sánchez SDC Gutiérrez-Ocejo RA Baylón-Pacheco L Pérez-Reyes OL. Electrolyzed oxidizing water modulates the immune response in BALB/c mice experimentally infected with *Trypanosoma cruzi* . Pathogens. (2020) 9:974. doi: 10.3390/pathogens9110974 33238401 PMC7700191

[31] Guedes PM Veloso VM Afonso LC Caliari MV Carneiro CM Diniz LF. Development of chronic cardiomyopathy in canine Chagas disease correlates with high IFN-gamma, TNF-alpha, and low IL-10 production during the acute infection phase. Vet Immunol Immunopathol. (2009) 130:43–52. doi: 10.1016/j.vetimm.2009.01.004 19211152

[32] Arce-Fonseca M González-Vázquez MC Rodríguez-Morales O Graullera-Rivera V Aranda-Fraustro A Reyes PA. Recombinant enolase of *Trypanosoma cruzi* as a novel vaccine candidate against Chagas disease in a mouse model of acute infection. J Immunol Res. (2018) 2018:8964085. doi: 10.1155/2018/8964085 29854848 PMC5964559

[33] Araujo Z El Bouhdidi A Heremans H Van Marck E Castés M Carlier Y. Vaccination of mice with a combination of BCG and killed *Leishmania* promastigotes reduces acute *Trypanosoma cruzi* infection by promoting an IFN-gamma response. Vaccine. (1999) 17:957–64. doi: 10.1016/s0264-410x(98)00311-9 10067703

[34] Arce-Fonseca M Gutiérrez-Ocejo RA Rosales-Encina L Aranda-Fraustro A Cabrera-Mata JJ Rodríguez-Morales O. Nitazoxanide: A drug repositioning compound with potential use in Chagas disease in a murine model. Pharmaceuticals. (2023) 16:826. doi: 10.3390/ph16060826 37375773 PMC10302963

[35] Espinoza B Rico T Sosa S Oaxaca E Vizcaino-Castillo A Caballero ML. Mexican Trypanosoma cruzi T. cruzi I strains with different degrees of virulence induce diverse humoral and cellular immune responses in a murine experimental infection model. J BioMed Biotechnol. (2010) 2010:890672. doi: 10.1155/2010/890672 20396398 PMC2852613

[36] Vizcaíno-Castillo A Jiménez-Marín A Espinoza B. Exacerbated skeletal muscle inflammation and calcification in the acute phase of infection by Mexican *Trypanosoma cruzi* DTUI strain. BioMed Res Int. (2014) 2014:450389. doi: 10.1155/2014/450389 24991553 PMC4060783

[37] Gorocica RPS Jiménez MMC Garfias BY Sad OI Lascurain R. Componentes glicosilados de la envoltura de *Mycobacterium tuberculosis* que intervienen en la patogénesis de la tuberculosis. Rev Inst Nal Resp Mex. (2005) 18:142–53.

[38] García-Hernández M Guerrero-Ramírez G Castro-Corona MÁ Medina-de-la-Garza CE. Inmunomodu-ladores como terapia adyuvante en la enfermedad infecciosa. Medicina Universitaria. (2009) 11:247–59.

[39] Chen J Gao L Wu X Fan Y Liu M Peng L. BCG-induced trained immunity: history, mechanisms and potential applications. J Transl Med. (2023) 21:106. doi: 10.1186/s12967-023-03944-8 36765373 PMC9913021

[40] Wong EC Angiono L Dorsch J Pintor L Blanco S Morral E. Pericarditis constructiva asociada a miocardiopatía chagásica. CONAREC. (2018) 33:322–24. doi: 10.32407/RCON

[41] Rodríguez-Morales O Mendoza-Téllez EJ Morales-Salinas E Arce-Fonseca M. Effectiveness of nitazoxanide and electrolyzed oxiding water in treating Chagas disease in a canine model. Pharmaceutics. (2023) 15:1479. doi: 10.3390/pharmaceutics15051479 37242721 PMC10224175

[42] Chaves AT Menezes CAS Costa HS Nunes MCP Rocha MOC. Myocardial fibrosis in Chagas disease and molecules related to fibrosis. Parasite Immunol. (2019) 41:e12663. doi: 10.1111/pim.12663 31309590

[43] Lima ES Andrade ZA Andrade SG. TNF-alpha is expressed at sites of parasite and tissue destruction in the spleen of mice acutely infected with Trypanosoma cruzi. Int J Exp Pathol. (2001) 82:327–36. doi: 10.1046/j.1365-2613.2001.00203.x PMC2517787 11846839

[44] Rosas F. Enfermedad de Chagas. Rev Colomb Cardiol. (2011) 18:241–44. doi: 10.1016/S0120-5633(11)70193-0

[45] Echavarría NG Echeverría LE Stewart M Gallego C Saldarriaga C. Chagas disease: chronic Chagas cardiomyopathy. Curr Probl Cardiol. (2021) 46:100507. doi: 10.1016/j.cpcardiol.2019.100507 31983471

[46] Verma A Pan Z. Chagas disease initially diagnosed in a lymph node. Blood. (2020) 136:2478. doi: 10.1182/blood.2020008815 33211840

[47] Bonney KM Luthringer DJ Kim SA Garg NJ Engman DM. Pathology and pathogenesis of Chagas heart disease. Annu Rev Pathol. (2019) 14:421–47. doi: 10.1146/annurev-pathol-020117-043711 PMC7373119 30355152

[48] Soares MB de Lima RS Rocha LL Vasconcelos JF Rogatto SR dos Santos RR. Gene expression changes associated with myocarditis and fibrosis in hearts of mice with chronic chagasic cardiomyopathy. J Infect Dis. (2010) 202:416–26. doi: 10.1086/653481 PMC2897928 20565256

[49] Torres-Castro M Hernández-Betancourt S Torres-León M Puerto F. Lesiones histológicas asociadas a la posible infección por *Trypanosoma cruzi* (Chagas, 1909) en corazones de roedores sinantrópicos capturados en Yucatán, México. Anales Biología. (2016) 38:29–35. doi: 10.6018/analesbio.38.03

[50] Ucan-Euan F Hernández-Betancourt S Arjona-Torres M Panti-May A Torres-Castro M. Estudio histopatológico de tejido cardiaco de roedores infectados con *Trypanosoma cruzi* capturados en barrios suburbanos de Mérida, México. Biomédica. (2019) 39:32–43. doi: 10.7705/biomedica.v39i3.4192 31529832

[51] Fontanella GH Pascutti MF Daurelio L Perez AR Nocito AL Wojdyla D. Improved outcome of *Trypanosoma cruzi* infection in rats following treatment in early life with suspensions of heat-killed environmental *Actinomycetales* . Vaccine. (2007) 25:3492–500. doi: 10.1016/j.vaccine.2006.11.062 17368877

[52] Marinho CR Bucci DZ Dagli ML Bastos KR Grisotto MG Sardinha LR. Pathology affects different organs in two mouse strains chronically infected by a *Trypanosoma cruzi* clone: a model for genetic studies of Chagas' disease. Infect Immun. (2004) 72:2350–57. doi: 10.1128/IAI.72.4.2350-2357.2004 PMC375186 15039360

[53] Vizcaíno-Castillo A. Respuesta inmune en órganos blanco infectados con cepas mexicanas de *Trypanosoma cruzi* . Ciudad de México: Universidad Nacional Autónoma de México, Facultad de Química (2007).

