# Supplementary material for: Mycobacterium bovis BCG as immunostimulating agent prevents the severe form of chronic experimental Chagas disease
Source: Front Immunol. 2024 Mar 21;15:1380049. doi: 10.3389/fimmu.2024.1380049 (PMC10991741; doi:10.3389/fimmu.2024.1380049)
Supplement: Supplementary file 1 [file DataSheet_1.pdf]

Supplementary Material

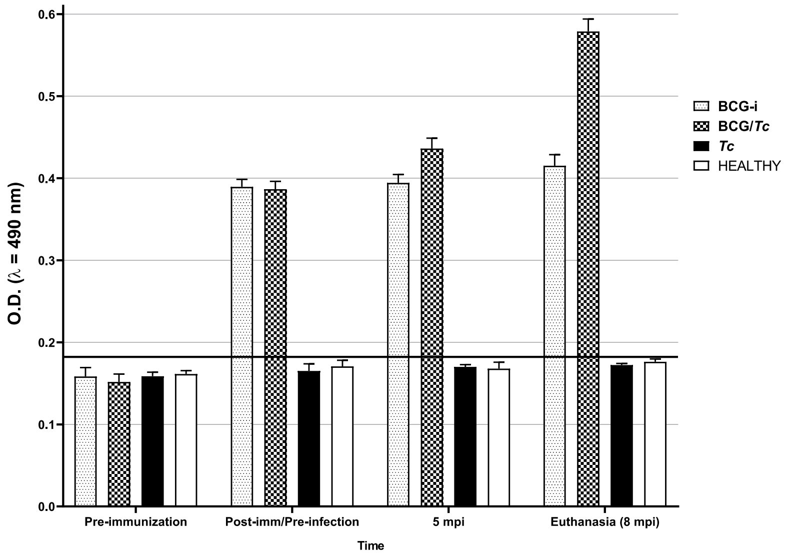

Supplementary Figure 1. Anti-IgG reactivity to bovine PPD from BCG-immunized or nonimmunized mice and infected or not with *T. cruzi*. Values of each group are representative of two independent experiments with equivalent results. Data are presented as mean with S.D. from individual $O.D._{450nm}$ values from each time. A One-Way ANOVA followed by Tukey´s multiple comparison test was used, and significant difference is shown (*) when $P \leq 0.05$. Black line shows the cut-off value. Post-imm = post-immunization.

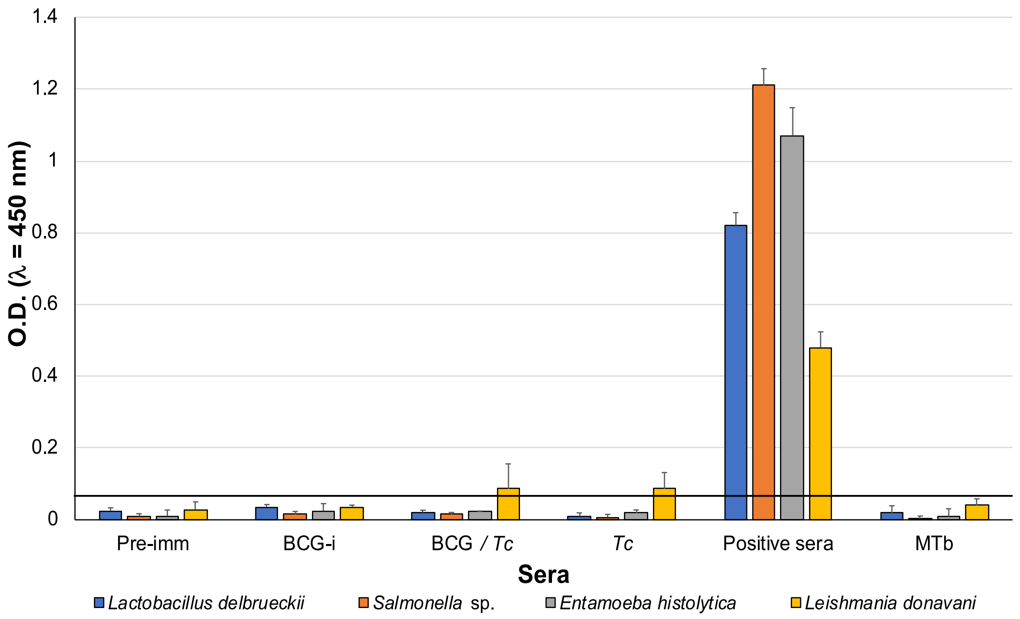

Supplementary Figure 2. Anti-IgG reactivity to different antigens from four microorganisms in BCG-immunized or nonimmunized mice and infected or not with *T. cruzi*. Values of each group are representative of two independent experiments with equivalent results. Data are presented as mean with S.D. from individual O.D.$_{450nm}$ values from each group. Black line shows the cut-off value. Pre-imm = Pre-immunization, MTb = *Mycobacterium tuberculosis* (serum from infected mice with Mtb).

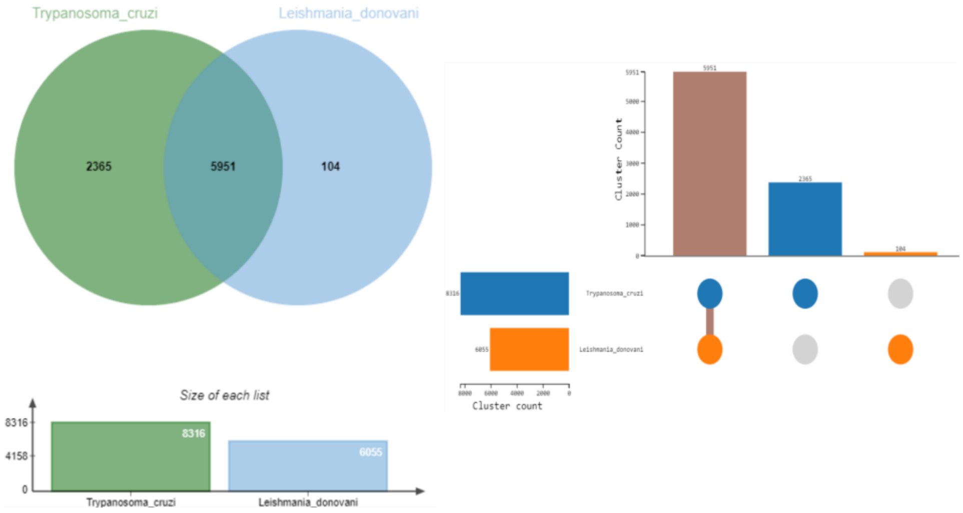

Supplementary Figure 3. Bioinformatic analysis by OrtoVenn 3 Program. Venn diagram shows the distribution of shared orthologous clusters between the two species: *Leishmania donovani* and *Trypanosoma cruzi*.

A B

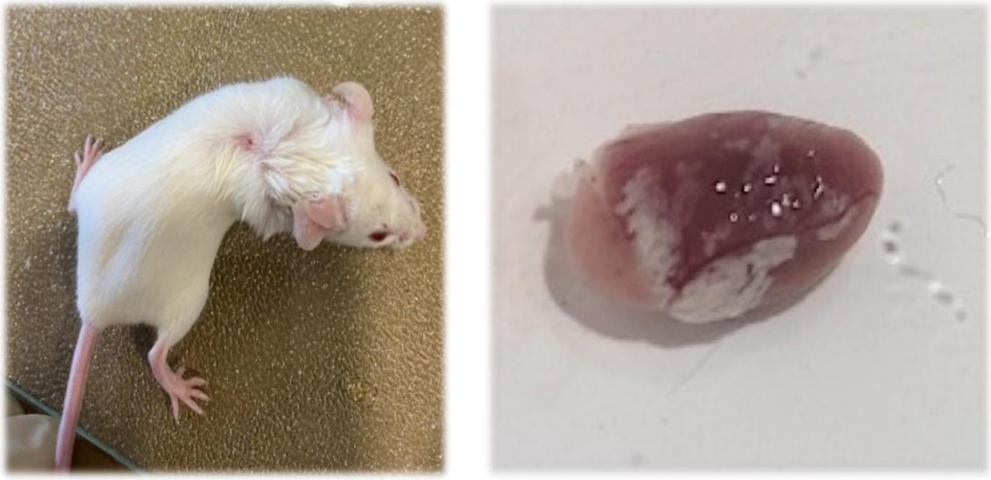

Supplementary Figure 4. Macroscopic lesions in BCG-immunized mice and infected with *T. cruzi*. (A) Alopecic lesion at the BCG inoculation site. (B) White/yellowish area on the surface of the heart in a mouse from the *Tc* group.
